# Evaluating Human Perception Thresholds in Magnetic Stimulation Using Experimental Measurements and Modelling

**DOI:** 10.1002/bem.70066

**Published:** 2026-07-27

**Authors:** Otto Kangasmaa, Ilkka Laakso

**Affiliations:** ^1^ Department of Electrical Engineering and Automation Aalto University Espoo Finland

**Keywords:** damped sinusoidal pulse, dosimetric modelling, induced electric field, method of adjustment, safety guidelines

## Abstract

This preregistered study investigated whether induced electric fields alone could predict human perception thresholds in magnetic stimulation. Using a figure‐of‐eight magnetic coil placed on the forearm, perception thresholds were collected from 24 healthy participants using the method of adjustment in 12 stimulation conditions. Subject‐fitted, anatomically realistic forearm models were constructed from a set of 10 MRI‐based models. Induced electric fields were computed using the finite element method with probabilistic tissue conductivities and post‐processed using spatial averaging and percentiles. Linear mixed‐effects modelling showed that the perception threshold was 32.2 V/m (SD between subjects 5.2 V/m) for a damped sinusoidal magnetic pulse with 83 μs phase duration. This threshold value was determined by spatial averaging over a cube with a side length of 2 mm and taking the 99th percentile of the electric field within the modelled forearms. However, the induced electric field did not fully explain the perception threshold (adjusted $R^{2}$ = 0.48; permutation testing, *p* < 0.001). We also conducted a supplementary modelling study, retrospectively analysing previous literature results to allow for direct comparison with our findings. These results showed that simplified calculations of the threshold electric field, obtained by modelling the forearm as a uniform cylinder, need to be scaled by a factor of 1.4 to align with the 99th percentile electric field calculated with anatomically realistic models. Both our experimental results and the modelling provide data to inform the refinement of safety guidelines.

## Summary

- Perception thresholds to magnetic stimulation were measured in 24 subjects, and the induced electric field was computationally modelled using subject‐specific forearm models.
- A perception threshold of 32.2 V/m was established for 83 μs damped sinusoidal magnetic pulses.
- Results from previous studies need to be scaled by a factor of 1.4 to align with calculations based on the 99th percentile electric field within the modelled forearms.

## 1 Introduction

Exposure to low‐frequency magnetic fields induces electric fields inside the human body. If strong enough, these induced electric fields may activate voltage‐gated ion channels in the nervous system, triggering action potentials, which result in muscle contractions or sensory perceptions. However, stronger fields, exceeding the aforementioned perception threshold, can cause adverse health effects, including pain, stimulation of the central nervous system (at worst causing seizures), or even stimulation of cardiac tissue (possibly resulting in arrhythmia). To mitigate these effects, international guidelines (ICNIRP 2010) and standards (IEEE‐C95.1 2019) set exposure limits intended to protect both the general public and occupational workers.

Most research on peripheral nerve stimulation (PNS) has been motivated by safety considerations in medical applications (outside the scope of the ICNIRP guidelines and the IEEE standards). For instance, in magnetic resonance imaging (MRI), PNS represents a critical safety constraint because strong gradient magnetic fields are required for spatial encoding of the imaged tissue. Experimental works studying PNS thresholds, or magnetic perception thresholds, have reported their thresholds in terms of the rate of magnetic field switching (dB/dt), gradient excursion and switching time, or slew rate, of the coil (Nyenhuis et al. 2001; Zhang et al. 2003; Chronik and Ramachandran 2003). However, nerve activation is caused by the induced electric field, the distribution of which depends not only on coil design but also on the shape, size, and arrangement of conductive tissue. Thus, the electric field is usually considered a better metric for exposure since it is comparable across sources of exposure.

If the induced electric field is known, nerve and muscle excitation thresholds can be computed with electrostimulation models, which take into account the spatial and temporal characteristics of the electric field. Both ICNIRP and IEEE have utilised, at least to some extent, electrostimulation models to form their restrictions. Using electrostimulation models is, however, more challenging than just calculating the electric field, as it requires information on the locations and trajectories of the nerve fibres. Moreover, choosing the appropriate models has also been shown to cause large variations in the estimated thresholds (Reilly 2016; Soyka et al. 2025).

So, could the induced electric field by itself be used as a predictor of perception thresholds in magnetic stimulation? This would avoid the cumbersome process of detailed electrostimulation modelling. Only two experimental studies have reported perception thresholds directly in terms of the induced electric field (Bourland et al. 1996; Havel et al. 1997). In both studies, the electric field was estimated using a simple relation between the maximal circumference of the forearm and the strength of the applied dB/dt pulse. With major advances in computational modelling over the past two decades, this question can now be re‐investigated by combining experimental data with simulations.

This preregistered study (https://doi.org/10.17605/OSF.IO/RMW2D) combines anatomically realistic forearm models and computational modelling with experimentally derived magnetic perception thresholds from 24 participants. We test a single hypothesis: magnetic stimulation perception thresholds are inversely proportional to the induced electric field strength. Additionally, we use the developed models to re‐analyse the findings reported by Havel et al. (1997). This work is based on an extended abstract presented at BioEM 2025 conference (Kangasmaa and Laakso 2025).

## 2 Materials and Methods

### 2.1 Study Participants

Twenty‐four participants (15 male, 9 female, mean age ± SD = 35 ± 12, age range: 23–65), all self‐reported as healthy, volunteered in the study, as stated in the sampling plan of the preregistration. All participants gave their verbal consent for participation through a thorough set of questions. The study was approved by the Aalto University Research Ethics Committee (decisions D/2578/03.04/2025). All procedures were conducted in accordance with the Declaration of Helsinki.

### 2.2 Experimental Setup and Procedure

**Figure 1:**
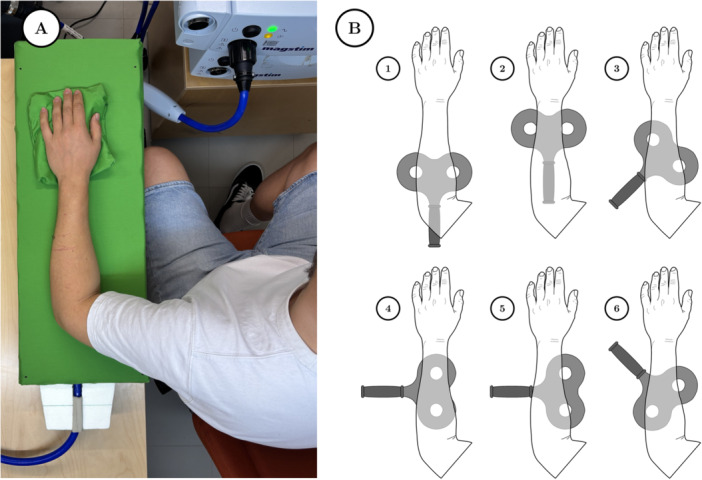
(A) Experimental setup with an example subject resting their left arm on a wooden box containing a figure‐eight magnetic coil. (B) The six approximate coil locations that were tested in the perception threshold experiments.

The perception threshold of participants due to magnetic stimulation was studied as described in the preregistration. Participants rested their left forearm on a wooden box containing a figure‐eight coil connected to a Magstim $200^{2}$ stimulator (Magstim Company, UK) (Figure 1A). The stimulator delivers a monophasic pulse, with the pulse shape dampened after the first quarter cycle (83 μs, see Supplementary Figure 1D in Laakso et al. 2025). The coil was positioned horizontally, 2.5 cm below the surface of the box, such that it was aligned in the same plane as the surface of the box.

With their right hand, the participants themselves adjusted the stimulation intensity and applied the magnetic pulses (method of adjustment). For the initial thresholds, participants were instructed to increase the stimulation intensity until a clearly perceptible sensation was felt, after which they adjusted it to their individual threshold level. This perception threshold was defined as the lowest intensity at which they could reliably perceive the stimulation, given as a percentage of the maximum stimulator output (MSO).

Six approximate coil locations were tested (Figure 1B). With each of these locations, the current direction was reversed, totalling 12 tested conditions. At each coil location, overhead images were taken to record the arm location with respect to the figure‐eight coil. Images were captured using the built‐in camera of an iPhone 15 Plus smartphone (Apple Inc., CA, USA) triggered by a Bluetooth shutter remote. The participants were asked to keep their left arm still when determining their perception threshold, but were allowed to move it freely between tests. The entire experimental session lasted roughly 30 min.

To control for potential order effects, a balanced Latin square design was employed for the coil location sequences. This design ensured that each of the six locations appeared equally often in each ordinal position and was preceded by every other condition an equal number of times. The six resulting sequences were evenly assigned across the sample of 24 participants (*n* = 4 per sequence). Within each coil location sequence, the two current directions were presented in one of four predefined orders (e.g., 1‐2‐1‐2‐1‐2…; 2‐1‐2‐1‐2‐1…; 1‐2‐2‐1‐1‐2…; or 2‐1‐1‐2‐2‐1‐…), ensuring that both directions were tested at each location while maintaining counterbalancing across participants.

### 2.3 Subject‐Fitted Forearm Models

**Figure 2:**
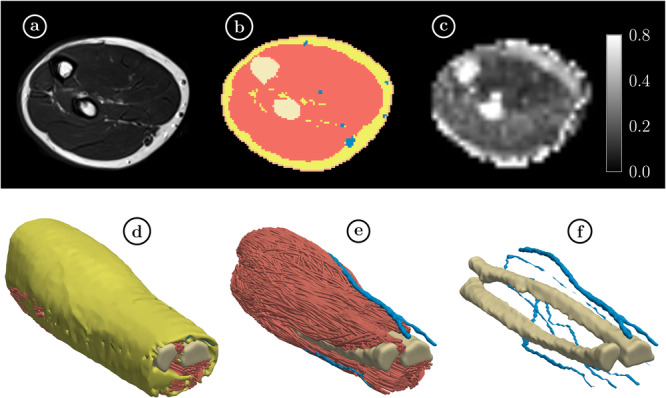
Data from an example forearm model. (a) T2‐weighted MRI; (b) segmented tissues; (c) fractional anisotropy in the axial plane; surface models of (d) fat, (e) muscle fibres, and (f) bone and blood.

To model the strength of the electric field induced in the subjects' forearms due to the magnetic stimulation, we used ten anatomically accurate forearm models from one of our previous works (Kangasmaa et al. 2025). These forearm models are based on MRI taken from six men and four women, and segmented into four different tissues: bone, fat, muscle and blood. Here, we voxelized these segmented forearms with a 1 mm × 1 mm × 1 mm resolution and added the skin onto the models as a 1 mm‐thick layer. An example T2‐weighted MR image and corresponding segmentation from the mid part of the forearm are shown in Figure 2a,b, respectively. Figure 2d–f shows surface models of fat, muscle, bone, and blood from the same forearm.

Directions of skeletal muscle fibres were estimated using diffusion‐weighted (DW) MRI. The DW‐MRI data had been acquired with 30 gradient orientations using $400{\text{s/mm}}^{2}$ diffusion weightings with a 3 mm × 3 mm × 3 mm voxel size. Four additional images without diffusion weightings had been acquired in the reverse‐phase encoding direction. The DW‐MR images were first corrected for susceptibility‐induced distortions (Andersson et al. 2003), eddy current‐induced distortions (Andersson and Sotiropoulos 2016), and low‐frequency intensity nonuniformities (Tustison et al. 2010) using the FIMRIB software library (Jenkinson et al. 2012). Finally, we estimated the diffusion tensor from the corrected DW‐MRI data using the MRtrix3 software package (Tournier et al. 2019). The resulting diffusion tensor is best visualised by calculating scalar maps, such as fractional anisotropy (Figure 2c), or by performing fibre tractography on the diffusion tensor (Aydogan and Shi 2021) and visualising the individual tracts (Figure 2e).

**Figure 3:**
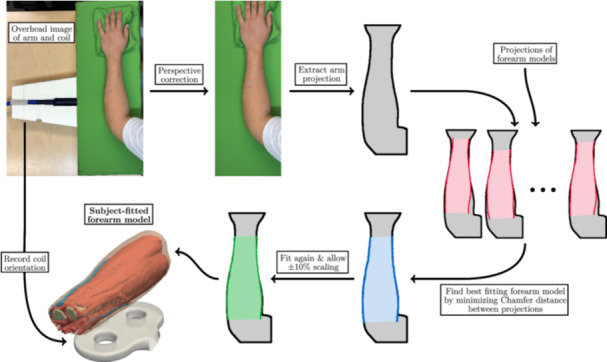
Workflow for generating subject‐specific forearm models from overhead images captured during perception threshold experiments. Perspective correction is performed using calibration points on the wooden box, and the coil's location and orientation are recorded. The arm outline is extracted and compared with projections of available gender‐specific MRI‐based forearm models, with the best fit found by minimising the Chamfer distance and further refined by ±10% isotropic scaling. This yields a subject‐fitted forearm model with an accurately defined coil position.

These forearm models, along with the overhead images captured during the perception threshold experiments, were used to create subject‐specific forearm models for each participant, with the coil accurately positioned relative to the forearm to replicate the experimental setup. For this, a custom programme was built in MATLAB 2022b (MathWorks Inc.). The process went as follows (Figure 3). First, the perspective of the camera was corrected with a projective transformation applied to the overhead images. This was done using known calibration points marked on the wooden box. Concurrently, the location and orientation of the coil were recorded. Second, the arm's outline was extracted utilising the green background. Third, the arm's outline was compared to projections of the forearm models. To find the best‐fitting model, we minimised the Chamfer distance between the two point clouds. Here, gender was taken into account, with males choosing from six available male models and females from four female models. Fourth, the best‐fitting model and the arm outline were once again fitted together, but this time, allowing ±10% isotropic scaling to ensure the best fit possible. Finally, a subject‐fitted forearm model was created with the coil position accurately defined. For the rest of the coil locations, we simplified this process by performing the perspective correction, only comparing the projection of this subject‐fitted model to the arm outline and recording the coil position.

### 2.4 Tissue Conductivities

The electrical conductivities for the forearm models' tissues were compiled from the literature. Rather than choosing a single value or taking a simple mean across multiple studies, we chose to construct probability distributions for the conductivity of each tissue. When choosing studies to consider, priority was given to studies that measured electrical properties in vivo, in human subjects, at body temperature, and low frequencies (≤10 kHz).

**Figure 4:**
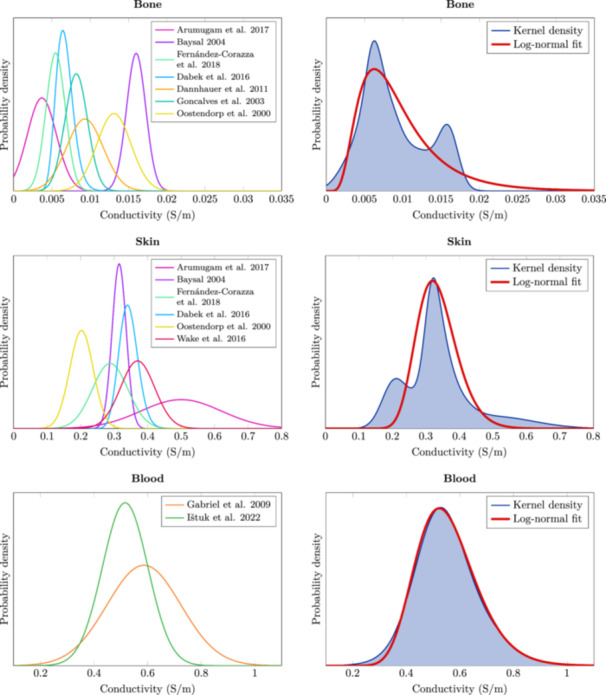
Left column: Tissue conductivities for bone, skin and blood compiled from literature. Right column: kernel density estimate of the compiled conductivity values with a fitted log‐normal distribution.

For bone, skin and blood, the compiled values are shown in the left column of Figure 4. The reported statistics from each study were used to construct normalised probability density functions. From these “kernels,” we constructed a kernel density estimate, shown in the right column of Figure 4. Since the individual kernels were treated as samples from the true probability distributions, and the conductivity cannot be negative, we fitted log‐normal distributions to the kernel density estimates.

The conductivities for fat and anisotropic skeletal muscle were taken from our previous study (Kangasmaa et al. 2025). There, we used a probabilistic approach to estimate their values over the low‐frequency range, yielding a probability distribution for the conductivity of fat and muscle parallel and perpendicular to muscle fibres. In the present work, the muscle fibre direction in each voxel was defined as the direction of the eigenvector corresponding to the largest eigenvalue of the estimated diffusion tensor. Voxels with a fractional anisotropy below 0.1 were considered isotropic and assigned an isotropic conductivity, which was calculated based on the anisotropic values.

### 2.5 Modelling the Induced Electric Field

At low frequencies, where the quasi‐static assumption is valid, the electric scalar potential equation for magnetic stimulation can be written as:

(1) $$\nabla \cdot \vec{\vec{\sigma }}\cdot \nabla \phi =-\nabla \cdot \vec{\vec{\sigma }}\frac{\partial A}{\partial t},$$

where ϕ is the electric scalar potential, $\vec{\vec{\sigma }}$ the anisotropic electric conductivity and A the magnetic vector potential induced by the coil. The magnetic vector potential was calculated by modelling the geometry of the coil (Supporting Information S1: Figure 1B in Laakso et al. (2025)) and the Biot‐Savart law. As a boundary condition, the normal component of the current density at the model surface is zero. Here, we numerically solved the scalar potential equation with an in‐house solver based on the finite element method (Laakso and Hirata 2012; Kataja et al. 2023). The induced electric field inside the forearms was then calculated using the gradient of the scalar potential and the magnetic vector potential:

(2) $$E=-\nabla \phi -\frac{\partial A}{\partial t}.$$

### 2.6 Data Processing and Statistical Analysis

#### 2.6.1 Preregistered Hypothesis

We hypothesise that the magnetic stimulation perception thresholds are inversely proportional to the induced electric field strength.

The induced electric field was calculated as described in Section 2.5. The conductivity values used for the forearm models were chosen as the mode of the fitted log‐normal probability distributions. These were: 0.006 S/m for bone, 0.32 S/m for skin, and 0.54 S/m for blood. For fat and muscle tissue, we used the maximum a posteriori estimate of the posterior probability distribution at 1 kHz. These were: 0.18 S/m for fat, 0.54 S/m longitudinal and 0.12 S/m lateral to muscle fibres. The isotropic muscle conductivity was calculated based on these values and was 0.30 S/m.

Since the induced electric field was numerically calculated, spatial averaging and percentiles were used to mitigate numerical artefacts (e.g. staircasing error). We used three different averaging cubes: 2 mm × 2 mm × 2 mm, 1 cm × 1 cm × 1 cm and 2 cm × 2 cm × 2 cm. For the 2 mm averaging cube, we then calculated the 100th, 99.9th, and 99th percentiles, and for the two larger cubes, only the 100th percentile. The spatial averaging was performed over the entire forearm model, ignoring tissue boundaries, slightly deviating from the averaging scheme described in the ICNIRP guidelines. Additionally, since the subject‐fitted models were allowed to be scaled by ±10% the averaging cubes were also scaled accordingly. Note that the electric field percentiles here are calculated within the forearm and are not directly comparable to whole‐body percentiles.

After calculating the electric field for all participants and coil locations, a linear mixed‐effect model (LMM) was then used to analyse the relationship between the perception threshold and the inverse of the electric field. The model's specification was ‘PT ~ −1 + inv$E^{n}$ + inv$E^{n}$:N + inv$E^{n}$:Gender + (−1 + inv$E^{n}$ + inv$E^{n}$:N ∣ Subject)’, where PT is the perception threshold, inv$E^{n}$ the inverse of the electric field, with n denoting the different averaging schemes and percentiles, and N the order of the coil location and current direction (1‐12, normalised to range −1 − +1). We used maximum likelihood estimation to fit model coefficients. We also tested for potential outliers, which were detected using the generalised extreme Studentised deviate test.

To determine which of the electric field averaging schemes and percentiles provides the best fit with the perception threshold, the different LMMs were compared with each other using a simulated likelihood ratio test (10,000 samples). The comparison of LMMs was also assessed using adjusted $R^{2}$ and log‐likelihood. Additionally, all models were compared with an alternative model in which the electric field was set to a constant, independent of the coil location ($E^{0}$). This was done to test whether the electric field improves fit beyond a null model where the perception threshold depends on the participant but not on the electric field; however, given the limited range of producible electric fields, this comparison is uninformative, so we report further exploratory analyses.

#### 2.6.2 Exploratory Analyses

The results of the preregistered analysis showed that none of the LMMs performed better than the alternative LMM. The reason why this alternative model resulted in a better fit was due to the relatively narrow ranges of induced electric field values and observed PTs. For instance, we could not use coil locations that would have induced very low electric field values, as those would have resulted in PTs above the MSO. Similarly, the maximum electric field values were limited by the distance between the coil and the arm, which could not be made closer than approximately 2.5 cm. The inclusion of such extreme values would have greatly worsened the fit of the alternative model and improved the fit of the LMMs with the electric field. Thus, any statistically significant differences in either direction would be artifacts, affected by the arbitrary exclusion or inclusion of extreme data points. Therefore, the original preregistered method to investigate the significance of the electric field was invalid.

Thus, to evaluate the robustness of our findings, we used permutation testing to assess the statistical significance of the LMMs. First, the perception thresholds of each subject were randomly rearranged (permutated) 10,000 times. Second, the LMM model coefficients were calculated with the permutated perception threshold. Finally, the adjusted $R^{2}$ was calculated for each permutated LMM (null distribution) and compared to the adjusted $R^{2}$ of the preregistered LMM.

### 2.7 Correcting Perception Thresholds From Havel et al. (1997)

In addition to our experimental measurements and modelling, we performed a supplementary modelling study to enable direct comparison with the results of Havel et al. (1997). In their study, Havel et al. stimulated the forearms of 14 volunteers with a solenoidal coil and estimated perception thresholds in terms of the induced electric field. However, they calculated the induced electric field with a simple relation between the maximal forearm circumference $\left(C_{arm}\right)$ and the strength of the applied dB/dt pulse:

(3) $$E=\left(\frac{C_{arm}}{4\pi }\right)\frac{dB}{dt}.$$

Therefore, we replicated their stimulation scenario and computationally modelled the induced electric fields, allowing us to derive a correction coefficient for their reported results.

The solenoidal coil used by Havel et al. was modelled according to their specifications. The coil's length was 7 cm with an inner radius of 5.7 cm with 74 turns of 10 AWG copper wire arranged in three layers. Considering the wire diameter (enamelled 10 AWG ≈ 2.75–3 mm), we arranged it with the mid‐layer having 24 turns and the top and bottom layers having 25. The longitudinal axis of the coil was aligned with the longitudinal axis of our forearm models, after which the coil was translated so that its centre was positioned at the location of maximum forearm girth.

When performing low‐frequency numerical dosimetry, the conductivity values assigned to model tissues represent a considerable source of uncertainty. An intuitive and simple way to determine how this uncertainty propagates into the induced electric fields is to use random sampling. Here, we drew 10,000 random samples from the log‐normally fitted probability distributions (bone, skin, and blood) and the posterior probability distribution (fat and anisotropic muscle). The induced electric field was then calculated with each sampled conductivity combination using the same methods as described in Section 2.5. The averaging scheme and percentile were chosen based on the best‐fitting LMM. Finally, simple linear regression analysis was used to assess the relationship between the induced electric field and maximal forearm girth and derive the correction coefficient.

## 3 Results

The results of the preregistered analyses are reported first, followed by the results of exploratory analyses. Comparative results, not in the scope of the preregistration, are reported last.

### 3.1 Confirmatory: Magnetic Stimulation Thresholds

To test our preregistered hypothesis, we modelled the induced electric field for each subject in all coil locations. We then used a linear mixed‐effect model to study the relationship between the experimentally derived perception threshold and the modelled induced electric field. As mentioned in Section 2.6‐1, we studied five different post‐processing schemes for $E^{n}$. Each were fitted to a model that was in the form:

(4) $$PT_{ij}=\frac{\alpha +A_{j}+\left(\beta +B_{j}\right)N_{i}+\gamma G_{i}}{E_{ij}^{n}}+\epsilon_{ij},$$

where i is the coil condition, j is the participant, $PT_{ij}$ is the perception threshold in terms of the MSO, $E_{ij}^{n}$ is $E^{n}$ calculated with the MSO, $N_{i}$ the order of the coil condition (scaled to [‐1,1]), $G_{i}$ is the gender of the subject (categorical: male or female), and α,β and γ are their fixed effect coefficients, respectively. The subject‐specific coefficients $A_{j}$ and $B_{j}$ and the residual error term $\epsilon_{ij}$ all follow normal distributions with zero mean and standard deviations of $\sigma_{A},\sigma_{B}$, and $\sigma_{\epsilon }$, respectively.

As stated in the preregistration, we compared the LMM with different averaging schemes and percentiles to each other with the simulated likelihood ratio test. The results showed that the models were always significantly better (p << 0.05) if their log‐likelihood was larger than the other models. The comparison of the LMMs was also assessed using adjusted $R^{2}$, which showed similar behaviour. These statistics are compiled in Table 1. The alternative LMM (invalid) is also included in the table. In all of the LMMs, no statistical significance was observed with the order of the coil locations and current directions. Similarly, gender was also not statistically significant in any of the tested LMMs.

**Table 1:** The five studied LMMs arranged from best to worst and the alternative LMM where the electric field is constant.

LMM	Averaging cube	Percentile	Adjusted $R^{2}$	Log‐likelihood
$E^{1}$	2 mm	99th	0.4753	380.3
$E^{2}$	2 mm	99.9th	0.4693	372.6
$E^{3}$	1 cm	100th	0.4227	361.2
$E^{4}$	2 mm	100th	0.4080	357.2
$E^{5}$	2 cm	100th	0.2847	335.1
$E^{0}$			0.5954	407.3

**Figure 5:**
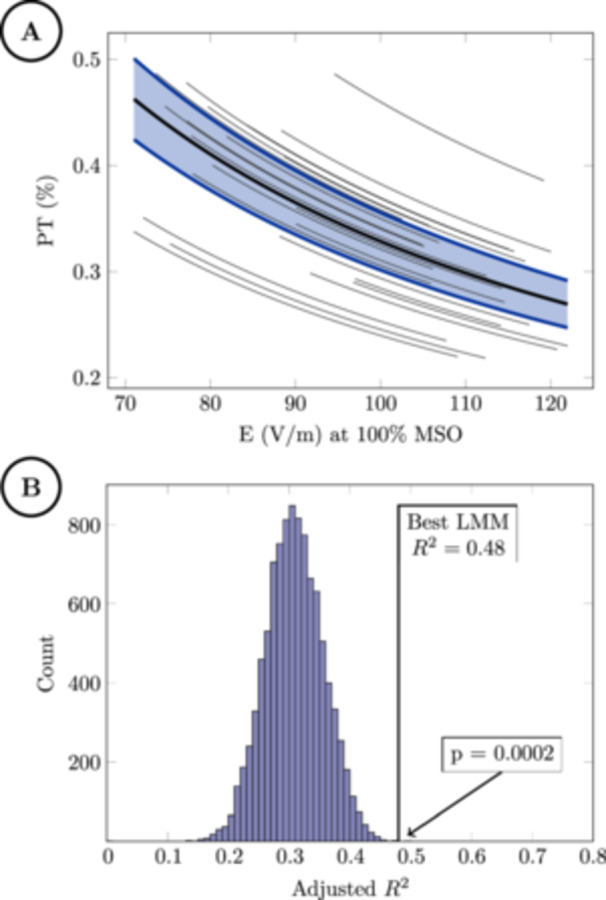
(A) Preregistered. The relationship between the perception threshold (PT) and the electric field ($E^{1}$). The black lines show the individual subject predictions from the LMM and the coloured region shows the predicted mean and 95% confidence intervals. (B) Exploratory. Permutation testing, with 10,000 random permutations.

The best fitting LMM was found when we used a 2 mm averaging cube, followed by taking the 99th percentile of the electric field. The coefficients of this LMM are shown in Table 2, and the rest of the LMMs can be found in the supplementary materials (supp_LMMdata.xlsx). According to the model, the mean predicted perception threshold is 32.2 V/m, with large between‐subject variability (standard deviation of 5.2 V/m). The predicted relationship between the perception threshold and the electric field is also shown in Figure 5A.

**Table 2:** Coefficient of best fitting LMM (2 mm averaging cube and 99th percentile).

Fixed effects			
Parameter	Estimate	95% CI	*p*‐value
α	32.2 (V/m)	29.9–34.4	4.22$\;\times \;10^{-84}$
β	0.104	−1.29–1.50	0.884
γ	−0.692	−2.80–1.42	0.519
Random effects			
$\sigma_{A}$	5.22 (V/m)	3.82–7.13	
$\sigma_{B}$	2.35	1.21–4.57	
$\sigma_{\epsilon }$	0.0572	0.0523–0.0626	

### 3.2 Exploratory: Permutation Testing

To test whether the effect of the electric field on the PT was statistically significant, we performed permutation testing with 10,000 random permutations. If there is an effect of the electric field, the LMM should fit better with real data than with randomly permuted data. With all preregistered LMMs, the adjusted $R^{2}$ was statistically significant compared to the permuted null distribution (*p*‐value < 0.05). Figure 5B shows the null distribution for the best‐fitting LMM.

### 3.3 Corrected Perception Thresholds From Havel et al. 1997

**Figure 6:**
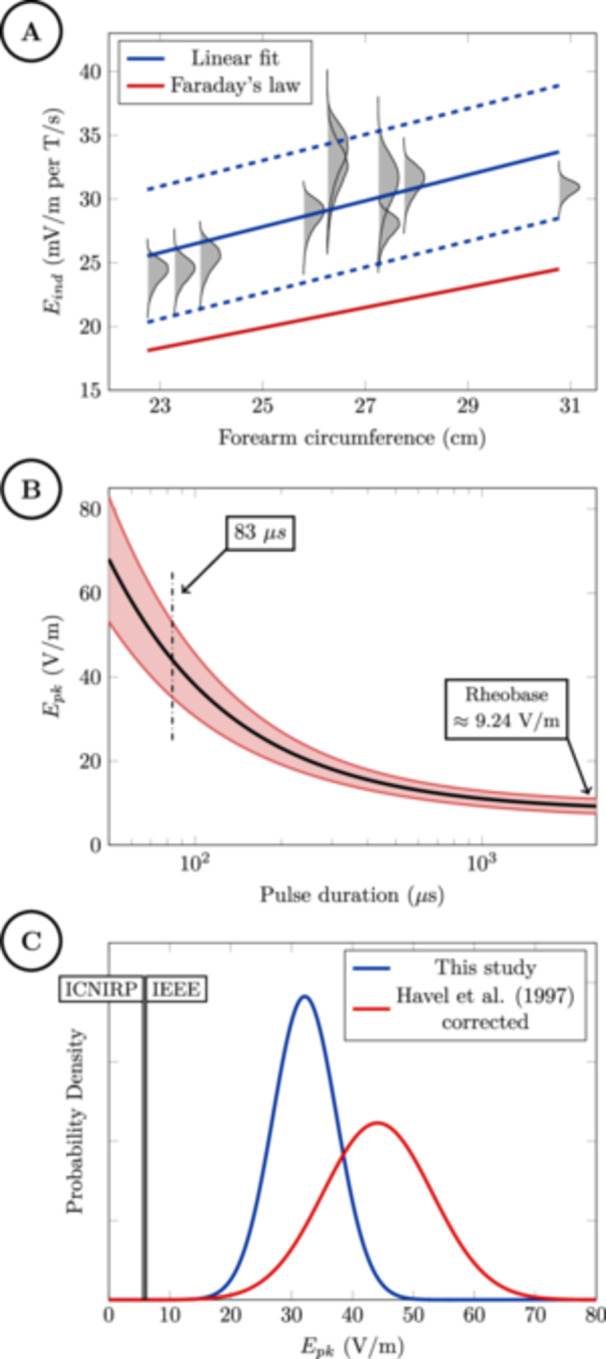
(A) Induced electric field due to a 1 T/s plotted as a function of maximal forearm girth. The grey distributions show how the electric field varies as a result of randomly sampling tissue conductivities. The blue lines show the linear regression line and 95% confidence intervals fitted to these electric field values, and the red line shows the electric field computed with Equation (3). (B) Corrected perception thresholds for damped sine pulses from Havel et al. (1997). (C) Perception threshold for a damped sine pulse of 83 μs. The threshold values used by ICNIRP and IEEE‐ICES are also shown for comparison.

Our supplementary modelling study showed that calculating the induced electric field with Faraday's law from the forearm circumference and dB/dt underestimates the induced electric field compared to the 2 mm averaged 99th of the electric field calculated with anatomically realistic models. The results are shown in Figure 6A where the induced electric field due to a time‐varying magnetic field of 1 T/s is plotted against the maximum girth of the forearm models. The grey half‐violin plots show how the electric field for each forearm is distributed due to the random sampling of the tissue conductivities. The linear regression line and corresponding prediction intervals are shown in blue, and the red line shows the relationship between the forearm circumference and the applied dB/dt pulse calculated with Equation (3). Thus, to convert the simplified electric field values of Havel et al. to 99th percentile electric field values, they need to be scaled with a correction coefficient:

(5) $$\text{coef(C)}=\frac{101.7C+2.381}{72.10C+2.302},$$

where C (m) is the maximum circumference of the forearm. Note that Havel et al. reported perception thresholds for both rectangular and damped sine pulses. This correction coefficient is valid for the latter. The correct values are shown in Figure 6B with the coloured region showing ± standard deviation. The perception thresholds from this experimental study and the corrected results from Havel. et al. are shown in Figure 6C.

## 4 Discussion

In this preregistered study, we investigated whether the induced electric field could be used to estimate the magnetic stimulation perception threshold. The perception threshold was found to be 32.2 V/m with a standard deviation of ±5.2 V/m between subjects. These values are valid for a damped sine magnetic pulse of 83 μs. The computationally derived electric field, which best explained the perception threshold, was found by averaging the calculated electric field in the forearms with a 2 mm×2 mm×2 mm cube, followed by taking the 99th percentile value. Our findings, however, only partially explain our hypothesis since the induced electric field only explained about 50% of the variance in the perception threshold.

Additionally, we estimated and applied a correction coefficient to the perception thresholds reported by Havel et al. (1997). Using the same formula as Havel et al. (Equation (3)) and our correction coefficient (Equation (5)), we can convert and correct perception thresholds from previous experimental works into thresholds that are in terms of the induced electric field. We included all experimental studies with a similar exposure condition (coil encircling a limb) (McRobbie and Foster 1984; Bourland et al. 1996; Saritas et al. 2013). The comparison is summarised in Table 3.

**Table 3:** Comparison of magnetic stimulation perception thresholds in terms of the 99th percentile induced electric field, obtained using correction coefficient (Equation (5)).

Study	N	Coil	Stimulus waveform	Location	Threshold	95% CI
					μ±σ (V/m)	(V/m)
This study	24	Figure‐8	Damped sine (83μs)	Forearm	32.9±5.2	30–34
Havel et al. (1997)	14	Solenoid	Damped sine (83μs)	Forearm	44.1±9.0	39–49
McRobbie and Foster (1984)	10	Helmholtz	Damped sine (83μs)	Forearm	46.1±6.3	44–48
Bourland et al. (1996)	10	Cylindrical	Damped sine (83μs)	Forearm	45.8±16	34–58
	10	Cylindrical	Damped sine (83μs)	Forearm	75.4±36 *	49–100
Saritas et al. (2013)	20	Solenoid	Sine wave (3 kHz)	Forearm	30.2±8.8	26–35
	17	Solenoid	Sine wave (3 kHz)	Leg	28.9±5.1	26–31

* Muscle activation measured from finger movement.

Several factors may account for the varying thresholds found in Table 3. The correction coefficient may have been overestimated. However, the use of 2‐mm spatial averaging combined with the 99th percentile provides a conservative adjustment. Also, anatomical variation between study populations is a possible factor, but is unlikely to fully explain the discrepancy. A more plausible explanation lies in how perception thresholds were defined. These earlier studies employed the method of limits and method of fixed intensities, whereas our participants were able to refine and converge on their threshold perception through repeated testing. This methodological difference likely contributed to the systematically lower thresholds in our study. Although our values differ from those reported previously, they provide complementary insight into the perception of magnetic stimulation when expressed in terms of the induced electric field.

Our experimental procedure included several potential confounding factors that may have influenced the perception threshold results. Discharge of the stimulation coil was accompanied by an abrupt clicking noise, which was not mitigated in this study. Psychological confounders were also present. The main one was the subjective determination of the lowest reliably perceived stimulation intensity, which participants may have interpreted differently. Since intensity was displayed as %MSO, participants could be influenced by specific values, and when thresholds fell between two values, personal preference may have led them to choose higher or lower options. Their approach to the threshold (from high to low or low to high) could also have affected responses. These factors, however, were accounted for using subject‐specific random effects in our linear mixed‐effect models. Recoskie et al. (2010) also found that perceptually derived thresholds correlated well with EMG measurements when they stimulated the ulnar nerve with both electric and magnetic stimulation, establishing the former as a simple and reliable measurement tool. Here, some of these confounding factors were intentionally left unmitigated, with the aim of keeping the setup and procedure simple, fast, accessible and non‐threatening, particularly as most participants were unfamiliar with such magnetic stimulation.

Since the induced electric field alone explained only 50% of the variation in the perception thresholds, electrostimulation models should be used to provide a more accurate representation of peripheral nerve activation during magnetic stimulation. However, because the forearm models do not include nerves, the reported thresholds should be interpreted as macroscopic field levels associated with perception, not as nerve‐specific activation thresholds. In this work, peripheral nerves were not included in our computational models because they were not visible in the original MR images. A possible approach would be to approximate peripheral nerve trajectories using external anatomical resources and incorporate them into our models. Potential sources include existing models, which include the peripheral nervous system (e.g., the male model by Zygote, American Fork, UT, USA, or the Yoon‐sun model from the Virtual Population Gosselin et al. (2014)), and detailed imaging datasets such as the Visible Human Project (U.S. National Library of Medicine 2023). With the nerve trajectories defined, the electric field data computed in this study could be projected onto these modelled nerve fibres and used to simulate nerve membrane dynamics. This approach could provide a more physiologically grounded framework for investigating perception thresholds in magnetic stimulation.

A question we have yet to answer is, why determine perception thresholds when, from a safety perspective, the goal is to prevent adverse health effects? The ICNIRP (2010) guidelines state that their primary objective is to “establish guidelines for limiting EMF exposure that will provide protection against adverse health effects.” Similarly, IEEE‐C95.1 (2019) standard identifies concerns such as “adversive or painful stimulation of sensory or motor neurons” and “muscle excitation that can lead to injury while performing potentially hazardous activities.” The perception thresholds derived in this study, both from our experimental data and from values corrected from Havel et al. were obtained using pulsed stimulation. In neither study did any participant report discomfort of any kind. Perception is not an adverse health effect. However, it is important to consider whether the same would hold true for continuous stimulation. In our view, continuous activation of sensory or motor neurons should be regarded as adverse, something that should not occur in daily life or in any occupational setting. From this perspective, perception thresholds offer practical and conservative safety limits: if exposure levels remain below the threshold for perception, adverse health effects are avoided with an additional margin of safety.

This work provides valuable insights into human perception thresholds to magnetic stimulation, which can help inform and refine safety guidelines. However, several important limitations related to both model development and experimental procedures should be addressed in future studies. First, the induced electric field may not be the most appropriate metric for exposure. Incorporating peripheral nerves into computational models would enable the use of electrostimulation models, thereby providing more accurate estimates of perception thresholds. Second, if additional measurements are performed, the experimental procedure should employ more robust methods to minimise the influence of potential confounding variables. Alternatively, perception thresholds could be assessed using physiological markers such as EMG. Finally, future studies could seek to identify the threshold for painful stimulation, for instance, by using long continuous waveforms or applying pulses with higher intensities.

## 5 Conclusion

This study explored the use of induced electric fields as predictors of perception thresholds during magnetic stimulation. By combining experimental data with computational models, we identify a perception threshold of 32.2 V/m with subject variability of 5.2 V/m for damped sinusoidal pulses of 83 μs. Our corrections to previous threshold estimates further highlight the need for additional experimental data and underscore the importance of accurate computational modelling.

## Ethics Statement

All participants gave their verbal consent for participation through a thorough set of questions, and the study was approved by the Aalto University Research Ethics Committee (diary number D/2578/03.04/2025).

## Conflicts of Interest

The authors declare no conflicts of interest.

## Preregistration

This study was preregistered on the Open Science Framework (https://doi.org/10.17605/OSF.IO/RMW2D).

## Supporting information


Supporting File

## Data Availability

The data that support the findings of this study are available from the corresponding author upon reasonable request.

## References

[bem70066-bib-0001] Andersson, J. L. , S. Skare , and J. Ashburner . 2003. “How to Correct Susceptibility Distortions in Spin‐Echo Echo‐Planar Images: Application to Diffusion Tensor Imaging.” NeuroImage 20, no. 2: 870–888. 10.1016/S1053-8119(03)00336-7. 14568458

[bem70066-bib-0002] Andersson, J. L. , and S. N. Sotiropoulos . 2016. “An Integrated Approach to Correction for off‐Resonance Effects and Subject Movement in Diffusion Mr Imaging.” NeuroImage 125: 1063–1078. 10.1016/j.neuroimage.2015.10.019. 26481672 PMC4692656

[bem70066-bib-0003] Arumugam, E. M. E. , S. Turovets , N. Price , D. Rech , P. Luu , and D. Tucker . 2017. “ In Vivo Estimation of Scalp and Skull Conductivity Using Beit For Non‐invasive Neuroimaging And Stimulation.” In Brain Stimulation and Imaging Meeting, Vancouver, BC.

[bem70066-bib-0004] Aydogan, D. B. , and Y. Shi . 2021. “Parallel Transport Tractography.” IEEE Transactions on Medical Imaging 40, no. 2: 635–647. 10.1109/TMI.2020.3034038. 33104507 PMC7931442

[bem70066-bib-0005] Baysal, U. , and J. Haueisen . 2004. “Use of a Priori Information in Estimating Tissue Resistivities–Application to Human Data In Vivo.” Physiological Measurement 25, no. 3: 737. 10.1088/0967-3334/25/3/013. 15253124

[bem70066-bib-0006] Bourland, J. , J. Nyenhuis , W. A. Noe , D. Schaefer , K. Foster , and L. Geddes . 1996. “ Motor and Sensory Strength‐Duration Curves for MRI Gradient Fields.” In Proceedings of the International Society for Magnetic Resonance in Medicine, New York, NY, 1723.

[bem70066-bib-0007] Chronik, B. A. , and M. Ramachandran . 2003. “Simple Anatomical Measurements Do Not Correlate Significantly to Individual Peripheral Nerve Stimulation Thresholds as Measured in MRI Gradient Coils.” Journal of Magnetic Resonance Imaging 17, no. 6: 716–721. 10.1002/jmri.10300. 12766901

[bem70066-bib-0008] Dabek, J. , K. Kalogianni , E. Rotgans , et al. 2016. “Determination of Head Conductivity Frequency Response In Vivo With Optimized eit‐eeg.” NeuroImage 127: 484–495. 10.1016/j.neuroimage.2015.11.023. 26589336

[bem70066-bib-0009] Dannhauer, M. , B. Lanfer , C. H. Wolters , and T. R. Knösche . 2011. “Modeling of the Human Skull in Eeg Source Analysis.” Human Brain Mapping 32, no. 9: 1383–1399. 10.1002/hbm.21114. 20690140 PMC6869856

[bem70066-bib-0010] Fernández‐Corazza, M. , S. Turovets , P. Luu , N. Price , C. H. Muravchik , and D. Tucker . 2018. “Skull Modeling Effects in Conductivity Estimates Using Parametric Electrical Impedance Tomography.” IEEE Transactions on Biomedical Engineering 65, no. 8: 1785–1797. 10.1109/TBME.2017.2777143. 29989921

[bem70066-bib-0011] Gabriel, C. , A. Peyman , and E. Grant . 2009. “Electrical Conductivity of Tissue at Frequencies Below 1 MHz.” Physics in Medicine and Biology 54, no. 16: 4863–4878. 10.1088/0031-9155/54/16/002. 19636081

[bem70066-bib-0012] Goncalves, S. , J. de Munck , J. Verbunt , F. Bijma , R. Heethaar , and F. LopesdaSilva . 2003. “In Vivo Measurement of the Brain and Skull Resistivities Using an Eit‐Based Method and Realistic Models for the Head.” IEEE Transactions on Biomedical Engineering 50, no. 6: 754–767. 10.1109/TBME.2003.812164. 12814242

[bem70066-bib-0013] Gosselin, M. ‐C. , E. Neufeld , H. Moser , et al. 2014. “Development of a New Generation of High‐Resolution Anatomical Models for Medical Device Evaluation: The Virtual Population 3.0.” Physics in Medicine & Biology 59, no. 18: 5287. 10.1088/0031-9155/59/18/5287. 25144615

[bem70066-bib-0014] Havel, W. , J. Nyenhuis , J. Bourland , et al. 1997. “Comparison of Rectangular and Damped Sinusoidal db/dt Waveforms in Magnetic Stimulation.” IEEE Transactions on Magnetics 33, no. 5: 4269–1271. 10.1109/20.619732.

[bem70066-bib-0015] ICNIRP . 2010. “Guidelines for Limiting Exposure to Time‐Varying Electric and Magnetic Fields (1 Hz–100 Khz).” Health Physics 99, no. 6: 818–836. 10.1097/HP.0b013e3181f06c86. 21068601

[bem70066-bib-0016] IEEE‐C95.1 . 2019. “IEEE Standard for Safety Levels With Respect to Human Exposure to Electric, Magnetic, and Electromagnetic Fields, 0 Hz to 300 GHz.” IEEE Std C95.1‐2019 (Revision of IEEE Std C95.1‐2005/ Incorporates IEEE Std C95.1‐2019/Cor 1‐2019) 0: 1–312. 10.1109/IEEESTD.2019.8859679.

[bem70066-bib-0017] Ištuk, N. , A. L. Gioia , H. Benchakroun , A. Lowery , B. McDermott , and M. O'Halloran . 2022. “Relationship Between the Conductivity of Human Blood and Blood Counts.” IEEE Journal of Electromagnetics, RF and Microwaves in Medicine and Biology 6, no. 2: 184–190. 10.1109/JERM.2021.3130788.

[bem70066-bib-0018] Jenkinson, M. , C. F. Beckmann , T. E. Behrens , M. W. Woolrich , and S. M. Smith . 2012. “Fsl.” NeuroImage 62, no. 2: 782–790. 10.1016/j.neuroimage.2011.09.015. 21979382

[bem70066-bib-0019] Kangasmaa, O. , and I. Laakso . 2025. “Computational Modelling of Human Forearm Perception Thresholds in Magnetic Stimulation [Conference Presentation].” BioEM 2025 Conference, Rennes.

[bem70066-bib-0020] Kangasmaa, O. , I. Laakso , and G. Schmid . 2025. “Estimating Human Fat and Muscle Conductivity From 100 Hz to 1 Mhz Using Measurements and Modelling.” Bioelectromagnetics 46, no. 1: e22541. 10.1002/bem.22541. 39825696 PMC11742663

[bem70066-bib-0021] Kataja, J. , J. Nissi , T. Roine , and I. Laakso . 2023. “Material Coarsening Strategy for Structured Meshless Multigrid Method for Dosimetry in Anisotropic Human Body Models.” IEEE Transactions on Electromagnetic Compatibility 65, no. 6: 1647–1655. 10.1109/TEMC.2023.3303533.

[bem70066-bib-0022] Laakso, I. , and A. Hirata . 2012. “Fast Multigrid‐Based Computation of the Induced Electric Field for Transcranial Magnetic Stimulation.” Physics in Medicine and Biology 57, no. 23: 7753–7765. 10.1088/0031-9155/57/23/7753. 23128377

[bem70066-bib-0023] Laakso, I. , J. Kataja , N. Matilainen , T. Roine , T. Tarnaud , and Y. Ugawa . 2025. “Locating Activation Sites of Tms With Opposite Current Directions Using Probabilistic Modelling and Biophysical Axon Models [Supplemental Material].” Brain Stimulation 18, no. 2: 215–224. 10.1016/j.brs.2025.02.003. 39955027

[bem70066-bib-0024] McRobbie, D. , and M. A. Foster . 1984. “Thresholds for Biological Effects of Time‐Varying Magnetic Fields.” Clinical Physics and Physiological Measurement 5, no. 2: 67–78. 10.1088/0143-0815/5/2/002. 6467871

[bem70066-bib-0025] Nyenhuis, J. A. , J. D. Bourland , A. V. Kildishev , and D. J. Schaefer . 2001. “Health Effects and Safety of Intense MRI Gradient Fields.” In Magnetic Resonance Procedures: Health Effects and Safety, edited by F. G. Shellock , 31–52. CRC Press, Boca Raton, FL.

[bem70066-bib-0026] Oostendorp, T. , J. Delbeke , and D. Stegeman . 2000. “The Conductivity of the Human Skull: Results of In Vivo and in Vitro Measurements.” IEEE Transactions on Biomedical Engineering 47, no. 11: 1487–1492. 10.1109/TBME.2000.880100. 11077742

[bem70066-bib-0027] Recoskie, B. J. , T. J. Scholl , M. Zinke‐Allmang , and B. A. Chronik . 2010. “Sensory and Motor Stimulation Thresholds of the Ulnar Nerve From Electric and Magnetic Field Stimuli: Implications to Gradient Coil Operation.” Magnetic Resonance in Medicine 64, no. 6: 1567–1579. 10.1002/mrm.22505. 20939088

[bem70066-bib-0028] Reilly, J. P. 2016. “Survey of Numerical Electrostimulation Models.” Physics in Medicine and Biology 61, no. 12: 4346. 10.1088/0031-9155/61/12/4346. 27223870

[bem70066-bib-0029] Saritas, E. U. , P. W. Goodwill , G. Z. Zhang , and S. M. Conolly . 2013. “Magnetostimulation Limits in Magnetic Particle Imaging.” IEEE Transactions on Medical Imaging 32, no. 9: 1600–1610. 10.1109/TMI.2013.2260764. 23649181

[bem70066-bib-0030] Soyka, F. , T. Tarnaud , C. Alteköster , et al. 2025. “Action Potential Threshold Variability for Different Electrostimulation Models and Its Potential Impact on Occupational Exposure Limit Values.” Bioelectromagnetics 46, no. 1: e22529. 10.1002/bem.22529. 39491315 PMC11650558

[bem70066-bib-0031] Tournier, J. ‐D. , R. Smith , D. Raffelt , et al. 2019. “Mrtrix3: A Fast, Flexible and Open Software Framework for Medical Image Processing and Visualisation.” NeuroImage 202: 116137. 10.1016/j.neuroimage.2019.116137. 31473352

[bem70066-bib-0032] Tustison, N. J. , B. B. Avants , P. A. Cook , et al. 2010. “N4itk: Improved n3 Bias Correction.” IEEE Transactions on Medical Imaging 29, no. 6: 1310–1320. 10.1109/TMI.2010.2046908. 20378467 PMC3071855

[bem70066-bib-0033] U.S. National Library of Medicine . 2023. The Visible Human Project. https://www.nlm.nih.gov/research/visible/visible_human.html. Accessed: August 18, 2025.

[bem70066-bib-0034] Wake, K. , K. Sasaki , and S. Watanabe . 2016. “Conductivities of Epidermis, Dermis, and Subcutaneous Tissue at Intermediate Frequencies.” Physics in Medicine and Biology 61, no. 12: 4376. 10.1088/0031-9155/61/12/4376. 27224275

[bem70066-bib-0035] Zhang, B. , Y. ‐F. Yen , B. A. Chronik , G. C. McKinnon , D. J. Schaefer , and B. K. Rutt . 2003. “Peripheral Nerve Stimulation Properties of Head and Body Gradient Coils of Various Sizes.” Magnetic Resonance in Medicine 50, no. 1: 50–58. 10.1002/mrm.10508. 12815678

